# Sleep Characteristics, Neighborhood Factors, Function, Mood, and Well-Being in Older Adults With Dementia

**DOI:** 10.1093/geroni/igaa057.2103

**Published:** 2020-12-16

**Authors:** Miranda McPhillips, Nancy Hodgson

## Abstract

The number of people with dementia is increasing worldwide. Circadian rhythm disorders and sleep problems are very common in this population and can have profound effects on well-being. Healthy Patterns Clinical Trial (NCT03682185) is a home-based activity intervention designed to improve circadian rhythm disorders and quality of life in people with dementia and their family caregivers. This symposium is designed to discuss the relationship between sleep characteristics and neighborhood environment, function, and psychological well-being in people with dementia. All analyses in this session were conducted on baseline data from participants enrolled in the Healthy Patterns Clinical Trial. We enrolled 170 individuals (67% female), aged (73.35 ± 8.74) with mean Clinical Dementia Rating (CDR) scores of (0.74 ± 0.51). Session 1 describes the role of neighborhood factors as influencing factors affecting sleep. Session 2 focuses on the relationship between sleep and mood. Session 3 focuses on the relationship between sleep and function. Session 4 focuses on the relationship between sleep and quality of life. Implications for future research and intervention development for people with dementia will be discussed.

